# Opportunities for Providing Web-Based Interventions to Prevent Sexually Transmitted Infections in Peru

**DOI:** 10.1371/journal.pmed.0040011

**Published:** 2007-02-27

**Authors:** Walter H Curioso, Magaly M Blas, Bobbi Nodell, Isaac E Alva, Ann E Kurth

## Abstract

The popularity of Internet cafes in Peru, even in poor communities with no modern infrastructure, opens new possibilities to develop online prevention and intervention programs for sexually transmitted diseases.

HIV is one of the biggest infectious killers worldwide, causing 8,000 deaths a day in 2005 [1]. In Latin America, an estimated 1.8 million people are living with HIV [1], and in 2005, about 66,000 people died of AIDS and 200,000 were newly infected with HIV [1].

In Peru, the HIV/AIDS epidemic has largely been concentrated among men who have sex with men (MSM) and female sex workers [2,3]. The seroprevalence rate for MSM is 10%–22% [3–5], compared to 0.1%–0.4% for the general population [6]. The prevalence of other sexually transmitted infections (STIs) among the MSM population is also high: 13.4% for syphilis and 46.3% for herpes simplex virus type 2 [3].

Even though the HIV/AIDS epidemic is confined to high-risk groups, there is significant risk for a wider HIV spread. A 2005 study in young males from socioeconomically disadvantaged populations showed that at least 14.2% of them had a male sexual partner in the last six months, and 86.3% of those who had a male sexual partner also had female partners in the same period [7]. Most of these young men's sexual encounters with male partners (56.9%) and female partners (84.2%) were unprotected [7].

## The Revolution of Internet Cafes (Cabinas Públicas) in Peru

One way of facilitating high-risk sexual encounters is through the Internet. In Peru, as in other cities of the developing world, Internet access is widely available through Internet cafes (cabinas públicas), small-scale storefront operations that offer low-cost and reliable connections. In Latin America, Internet cafes have been growing since around 1998, when competition in the telecommunications sector decreased the prices of dedicated phone lines [8].

In Peru, by February 2005, more than 10,000 cabinas públicas (at least 6,000 in Lima) were distributed throughout the country [9]. Peru is one of the countries with the highest number of Internet users in public places. By January 2006, there were an estimated 10 million Internet users in Peru [10].

**Figure 1:**
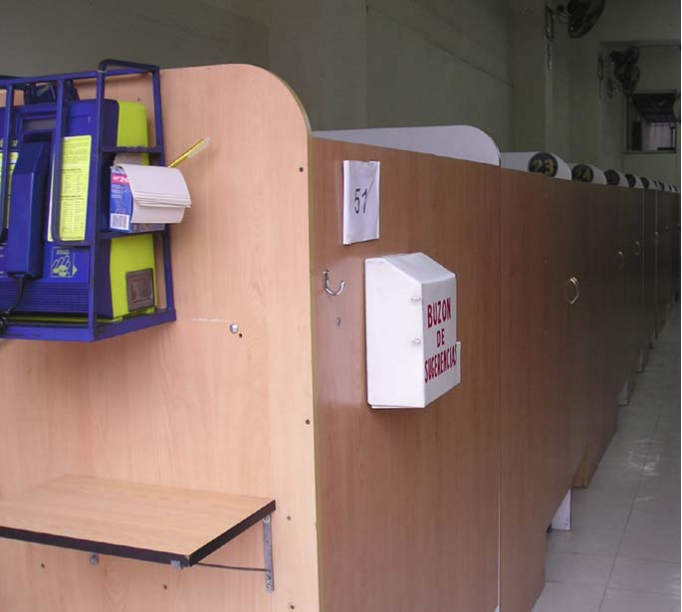
A typical cabina pública in Peru with private booths (Photo: Magaly Blas)

Cabinas are characterized by their low prices—an average of 15–30 cents (US) per hour—and relatively efficient connectivity [8,11]. The cabinas are the result of thousands of commercial initiatives of small informal entrepreneurs, and they offer other services as needed such as faxing, scanning, printing, photocopying, text editing, CD/DVD-writing, long-distance calls, and videoconferencing; some sell food and drinks (Figure 1). Cabinas are user-friendly and even unskilled users can rent a computer and, in most cases, receive basic assistance in operating computer programs [8]. Some are open 24 hours a day [12], and it is common to find cabinas even in poor urban slums.

In a 2000 national survey of 1,752 people (range 15–60 years, mean = 22.7 years) in five major Peruvian cities, 80.4% of Internet users (defined as those accessing the Internet at least once a week) said they accessed the Internet through cabinas públicas [13]. More recently, in a 2006 survey conducted by The APOYO Group, a business consulting firm based in Lima, 83% of Internet users in Lima (defined as those accessing the Internet at least once a month) accessed the Internet through cabinas públicas [14].

## Looking for Sex on the Internet

The wide pool of potential sex partners found online, coupled with ease of travel, are factors that could increase the spread of STIs [15,16], especially among MSM, for whom the Internet provides a new meeting venue for sex partners [17–19]. Recent studies show an association between very high-risk sexual behavior and meeting sex partners online [19,20]. MSM may find this virtual form of sex-seeking appealing because it offers anonymity, privacy, safety, minimal cost, and easy access at any time [21].

The ease of finding sex partners online increases STI and HIV risk, according to previous research [15,22,23]. Wong et al. reported that in San Francisco, having sex with an Internet partner is a risk factor for syphilis in MSM (odds ratio = 2.1 [95% confidence interval, 1.0–4.3]) [24]. The data available about this phenomenon are mainly limited to the United States [25–33] and other parts of the world such as the Netherlands [34,35], the United Kingdom [20,36–38], Sweden [39], and Canada [40,41]. An outbreak of syphilis in MSM in San Francisco, for example, was traced to an Internet chat room where the infected men had first met [15].

In Latin America, to our knowledge, only two studies have examined the role of the Internet in facilitating sexual contacts, and both were done in Peru. The first one, a study involving 100 HIV-positive patients who attended an HIV clinic in Lima, found that among men who reported seeking sex on the Internet, 94% were MSM and the remaining 6% were heterosexual (p = 0.032); all five respondents who reported having had sex with a partner found online were MSM [42].

The second study, by Blas et al., advertised an online survey through banner ads displayed on a popular Peruvian gay Web site [43]. The researchers received 1,124 completed surveys during the three months the survey was advertised. Cabinas públicas were reported as the main place of Internet access among 58% of the participants. Regarding Internet sex-seeking behaviors, 82% of those who reported that they were HIV negative or whose HIV status was unknown sought sex on the Internet, and 67% had sex with an Internet partner during the last year. Among 52 HIV-positive participants, 77% sought sex and 67% had sex with an Internet partner during the last year (personal communication, M. Blas, January 29, 2007).

## Cabinas Públicas as a Venue for Cybersex and Real Sex

A particular feature of some cabinas (Figure 1) is the availability of private modules so that clients can have sex on-site or arrange privacy for cybersex using Web cams to engage in sexual self-stimulation while online with another person. In the online survey conducted by Blas et al. [43], 1% (10/1,112) of the respondents (mean 26 years) reported having had their last sexual intercourse inside a private module of an Internet cafe. Of those who responded, nine out of ten had anal sex (only four used a condom), and one out of ten had oral sex without a condom. Of those who had anal sex, four out of nine had a casual partner, three out of nine an anonymous partner, and two out of nine a stable partner. All last sexual partners were males and all were met on the Internet.

Whether sexual encounters in cabinas are associated with HIV/STI incidence in Peru remains an open question that requires further investigation.

## Opportunities for Providing Web-Based Interventions

Given the possible association between HIV/STI transmission and the high level of Internet use by MSM in Peru, cabinas públicas are a logical place to deliver Web-based interventions. Cabinas also may be an effective means for delivering low-cost prevention messages to a great number of people, especially those who are not being reached using more traditional methods [43–45]. In Lima, traditional MSM outreach has been done face-to-face by peers in streets, discotheques, bathhouses, and bars.

Currently, two Peruvian institutions, Asociación Vía Libre and Asociación Civil Impacta conduct weekly counseling sessions by chat targeted to MSM from GayPeru.com (http://www.gayperu.com) (personal communication, M. Blas, January 29, 2007). A few people reported having had their last sexual intercourse inside a private module of an Internet cafe, according to our last survey (personal communication, M. Blas, January 29, 2007). It may be valuable to test the effectiveness of a prevention strategy that is based in the cabinas themselves. For example, cabina owners could be encouraged to sell or distribute condoms and display health referral information on HIV/STIs. The Web-based interventions also have the ability to reinforce STI information obtained in clinic settings among those who seek STI testing [46,47].

We are planning to carry out an Internet-based intervention for MSM to facilitate HIV/STI screening, early diagnosis, and treatment. Such strategies have been used in the US to increase access to STI testing [48–50], and in the Netherlands to negotiate safe sex practices among single gay men and their partners [51].

While such Web-based preventions are promising, three significant barriers remain: (1) It is unclear whether participants in Peru would use Web-based prevention interventions; (2) Some populations (e.g., those who are illiterate) may be hard to reach using Internet-based techniques; and (3) It is not proven whether Web-based prevention interventions would work in Peru (though there is evidence of their effectiveness in other parts of the world) [26,51–53].

## Conclusion

The unique popularity of cabinas públicas in Peru, even in poor communities with no modern infrastructure, opens new possibilities to develop and evaluate Web-based prevention and intervention programs for HIV/STIs. In theory, Web-based interventions can be delivered at low cost, and can be accessed by a large number of participants, and they might be a good way to target an MSM community that may not easily be reachable via traditional health campaigns. Future research should be undertaken to establish the acceptability and efficacy of Internet-delivered HIV/STI interventions in Peru and other resource-constrained settings.

## Supplementary Information

Alternative Language Abstract S1 Spanish translation of the abstract by IEA (19 KB DOC).

Figure S1 Screenshot of a random chat session in Spanish found at latinchat.com on November 2005. “Kenny,” a person from Lima, is looking for “actives” (insertive anal sexual partners).
(163 KB PPT).

## Abbreviations

MSM: men who have sex with men
STI: sexually transmitted infection
