# Supplementary material for: Opportunities for Providing Web-Based Interventions to Prevent Sexually Transmitted Infections in Peru
Source: PLoS Med. 2007 Feb 27;4(2):e11. doi: 10.1371/journal.pmed.0040011 (PMC1808078; doi:10.1371/journal.pmed.0040011)
Supplement: Alternative Language Abstract S1 — (19 KB DOC). [file pmed.0040011.sd001.doc]

# Resumen

En el Perú, el VIH y las infecciones de transmisión sexual (ITS) están concentrados principalmente en grupos de alto riesgo, tales como hombres que tienen sexo con hombres (HSH). La Internet está convirtiéndose en un método nuevo y popular que estos grupos usan para encuentros sexuales, muchos de los cuales son de alto riesgo para la transmisión de VIH/ITS. En el Perú, el acceso público a Internet es casi ubicuo a través de los café Internet llamados “cabinas públicas”. El Perú tiene uno de los números más grandes de usuarios de Internet en lugares públicos a nivel mundial. Muchas cabinas ofrecen acceso a computadoras con divisiones o dentro de habitáculos de tal modo que los clientes pueden tener total privacidad. Algunas personas pueden contactar parejas sexuales, mirar e intercambiar pornografía, tener sexo virtual usando cámaras web e involucrarse en actos sexuales reales. Debido al acceso masivo a Internet en el Perú, las intervenciones educativas y de prevención para las ITS, incluyendo el VIH, a través de la web, pueden ser un medio efectivo para ofrecer mensajes de prevención a grupos de alto riesgo. Estas intervenciones a través de la web pueden ser llevadas a cabo a bajo costo y pueden ser accesibles a un gran número de participantes. Discutimos sobre algunas oportunidades sobre el uso de los café Internet en estrategias de prevención para el VIH/ITS en el Perú.
