# Supplementary material for: Opportunities for Providing Web-Based Interventions to Prevent Sexually Transmitted Infections in Peru
Source: PLoS Med. 2007 Feb 27;4(2):e11. doi: 10.1371/journal.pmed.0040011 (PMC1808078; doi:10.1371/journal.pmed.0040011)
Supplement: Figure S1 — “Kenny,” a person from Lima, is looking for “actives” (insertive anal sexual partners). (163 KB PPT). [file pmed.0040011.sg001.ppt]

## Slide 1
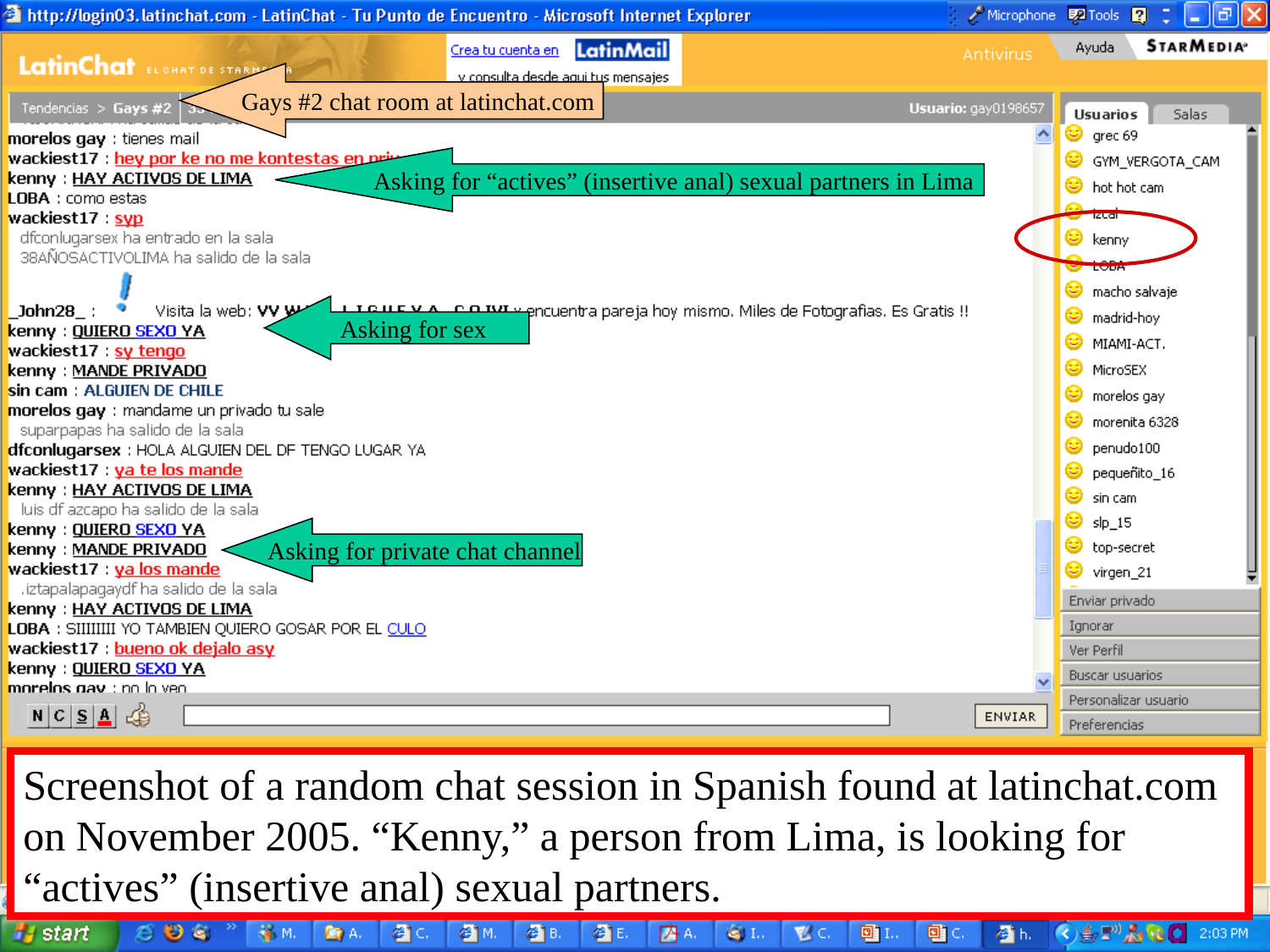

Gays #2 chat room at latinchat.com
#
Asking for “actives” (insertive anal) sexual partners in Lima
Asking for sex
Asking for private chat channel
Screenshot of a random chat session in Spanish found at latinchat.com on November 2005. “Kenny,” a person from Lima, is looking for “actives” (insertive anal) sexual partners.
